# Sex differences in the medial prefrontal cortical glutamate system

**DOI:** 10.1186/s13293-022-00468-6

**Published:** 2022-11-08

**Authors:** Melissa C. Knouse, Anna G. McGrath, Andre U. Deutschmann, Matthew T. Rich, Lia J. Zallar, Anjali M. Rajadhyaksha, Lisa A. Briand

**Affiliations:** 1 Department of Psychology, Temple University, Weiss Hall, 1701 North 13th Street, Philadelphia, PA 19122 USA; 2 Neuroscience Program, Temple University, Philadelphia, USA; 3 Department of Psychiatry, Robert Wood Johnson Medical School, Rutgers University, Piscataway, NJ 08854 USA; 4 Department of Pharmacology, Weill Cornell Medicine of Cornell University, New York, NY USA; 5 Pediatric Neurology, Pediatrics, Weill Cornell Medicine of Cornell University, New York, NY USA; 6 Feil Family Brain and Mind Research Institute, Weill Cornell Medicine of Cornell University, New York, NY USA; 7 Weill Cornell Autism Research Program, Weill Cornell Medicine of Cornell University, New York, NY USA

**Keywords:** Medial prefrontal cortex, Sex differences, AMPA receptors, Glutamate, Excitatory transmission

## Abstract

**Background:**

Dysregulation in the prefrontal cortex underlies a variety of psychiatric illnesses, including substance use disorder, depression, and anxiety. Despite the established sex differences in prevalence and presentation of these illnesses, the neural mechanisms driving these differences are largely unexplored. Here, we investigate potential sex differences in glutamatergic transmission within the medial prefrontal cortex (mPFC). The goal of these experiments was to determine if there are baseline sex differences in transmission within this region that may underlie sex differences in diseases that involve dysregulation in the prefrontal cortex.

**Methods:**

Adult male and female C57Bl/6J mice were used for all experiments. Mice were killed and bilateral tissue samples were taken from the medial prefrontal cortex for western blotting. Both synaptosomal and total GluA1 and GluA2 levels were measured. In a second set of experiments, mice were killed and ex vivo slice electrophysiology was performed on prepared tissue from the medial prefrontal cortex. Spontaneous excitatory postsynaptic currents and rectification indices were measured.

**Results:**

Females exhibit higher levels of synaptosomal GluA1 and GluA2 in the mPFC compared to males. Despite similar trends, no statistically significant differences are seen in total levels of GluA1 and GluA2. Females also exhibit both a higher amplitude and higher frequency of spontaneous excitatory postsynaptic currents and greater inward rectification in the mPFC compared to males.

**Conclusions:**

Overall, we conclude that there are sex differences in glutamatergic transmission in the mPFC. Our data suggest that females have higher levels of glutamatergic transmission in this region. This provides evidence that the development of sex-specific pharmacotherapies for various psychiatric diseases is important to create more effective treatments.

## Highlights

- Female mice exhibit heightened synaptosomal expression of AMPAR subunits GluA1 and GluA2 in the mPFC compared to males.
- Females have enhanced glutamatergic transmission in the mPFC compared to males.
- There are baseline sex differences in the mPFC glutamate system that may underlie sex differences in the prevalence and presentation of certain psychiatric diseases.

## Introduction

The prefrontal cortex (PFC) consists predominantly of pyramidal glutamatergic neurons [1] and acts as a driver of goal-directed behavior [2]. The medial PFC (mPFC) in particular is crucial for reward processing, attention, and memory [3]. The nature of its role in these processes has made it an interesting target for studies on psychiatric diseases involving dysregulated cognitive processing and motivation. Indeed, dysregulation in the mPFC is consistently implicated in illnesses including anxiety, depression, and substance use disorder (SUD) [3–8]. While the specific mechanisms driving various disease states differ, the mPFC is an important contributor to the presentation of these illnesses.

Imaging studies indicate that depressed patients have reduced mPFC volume compared to healthy control subjects [9, 10]. Further, it is proposed that individuals with generalized anxiety disorder may have elevated activation in the mPFC [11]. Additionally, smokers exposed to smoking-related cues exhibit increased activation in mPFC subregions, an effect that is modulated by smoking expectancy [12, 13]. There is also evidence that altering mPFC activity can impact symptomology in clinical populations. Continuous theta burst stimulation delivered to portions of the mPFC decreases drug cue reactivity in cocaine and heavy alcohol users and reduces craving in cocaine users [14, 15]. Altogether, these data indicate that the mPFC is an important contributor to the clinical presentation of psychiatric illnesses such as depression, anxiety, and SUD.

Biological sex was traditionally ignored as a variable in these illnesses [16, 17]. Despite this fact, there are notable sex differences emerging in the prevalence and presentation of disorders associated with mPFC dysfunction. Rates of depression and anxiety are higher in women than men [18–21]. The age of depression and anxiety onset is lower in females, and depressive episodes last longer and occur more frequently in women than men [22, 23]. There are established sex differences in SUD as well, with men being diagnosed more frequently, but women being more prone to drug craving [24–26]. Additionally, women relapse to drug use more readily than men, and men have longer periods of abstinence than women [27].

There are also sex differences in treatment efficacy for these illnesses. While there is still no clear consensus, clinical studies show that men and women likely do not respond in the same manner to the different classes of antidepressants [22]. For example, some studies show better therapeutic outcomes in women taking selective serotonin reuptake inhibitors (SSRIs) for depression, but men have better therapeutic outcomes with the tricyclic antidepressant imipramine [22]. However, there is also evidence fluoxetine, an SSRI, can be less effective in treating generalized anxiety disorder in women than men [23]. There are also emerging sex differences in the treatment outcomes of men and women undergoing buprenorphine maintenance for opioid use disorder, though more studies are needed [28]. Overall, these data indicate that biological sex likely influences treatment outcomes of psychiatric diseases that involve dysregulation in the PFC.

At baseline, biological sex and estrus cycle can influence electrophysiological properties of neurons within brain regions such as the striatum and PFC [29–32]. Alterations in glutamate signaling specifically may contribute to these sex differences in psychiatric disorders such as depression, anxiety and SUD [33]. Sex differences in levels of glutamate, the brain’s most prevalent excitatory neurotransmitter, are seen in several brain regions [34]. Several sex differences in the glutamatergic system have been observed, including differences in AMPA and NMDA receptor signaling, and differences in long-term potentiation [33, 35]. However, less is known about baseline sex differences in glutamatergic transmission specifically in the mPFC.

Glutamatergic transmission between the mPFC and other reward structures is implicated in a spectrum of psychiatric illnesses [4]. As there are known sex differences in psychiatric diseases involving the mPFC, we hypothesized there may be sex differences in glutamatergic transmission within this region that could drive these differences seen clinically. To determine this, we examined baseline sex differences in mPFC glutamate receptor expression and function. Our data indicate there are baseline sex differences in glutamatergic transmission within this region, with females exhibiting enhanced glutamatergic transmission in the mPFC compared to males.

## Materials and methods

### Subjects

33 male and female C57Bl/6J mice were bred in house for all experiments. Animals (8 weeks old) were group housed throughout the experiments with food and water available ad libitum. All animals were housed in a temperature- and humidity-controlled animal care facility. Mice had a 12-h light/dark cycle (lights on at 7:00 A.M.). Estrus cycle was not monitored in female animals during the course of these experiments. All procedures were approved by the Temple University Animal Care and Use Committee.

### Tissue processing and fractionation

Tissue samples were processed as previously published [36]. Briefly, bilateral mPFC tissue including the infralimbic and prelimbic regions (anterior–posterior 2.0, lateral ± 0.5, dorso-ventral − 1.5 to − 3.2) was dissected from 13 animals (7 females, 6 males). Tissue was then homogenized with a Teflon pestle (Pyrex) in 150 μl ice-cold sucrose buffer containing protease and phosphatase inhibitors. Homogenates were spun at 1000×*g* for 10 min at 4 °C. Forty μl of supernatant was saved for the total protein lysate fraction and the remainder was spun at 1000×*g* for 5 min 4 °C. The supernatant was then spun at 12,000×*g* for 20 min at 4 °C. The pellet was resuspended in 100 μl ice-cold Hepes/EDTA buffer containing protease and phosphatase inhibitors and spun at 12,000×*g* for 20 min at 4 °C. The pellet was then resuspended in 100 μl of HEPES/EDTA buffer containing protease and phosphatase inhibitors and saved as the synaptosomal protein lysate fraction. Protein concentration was measured using a BCA protein assay kit (Thermo Fischer Scientific).

*Western blot analysis.* 20–30 mg of protein was run on a 10% SDS-PAGE gel electrophoresis (constant 200 V, 50 min). Proteins were transferred to a PDVF membrane (constant 0.3 mA, 3 h) and transfer efficacy was verified with Ponceau S staining. Membranes were probed with primary antibodies against GluA1 (Abcam, ab140739, 1:1000), GluA2 (EMD, 07-261, 1:250), and GAPDH (Abcam, ab22555, 1:20000), and a peroxidase-labeled anti-rabbit secondary antibody (Vector, PI-1000, 1:5000). Signal was quantified using ImageJ analysis software (NIH). Protein quantities were normalized to GAPDH as a protein loading control.

*Slice preparation.* 20 animals (10 females, 10 males) were used for electrophysiology experiments. Mice were decapitated following cervical dislocation. The brain was removed and coronal slices (250 μm) containing the PFC were cut with a Vibratome (VT1000S, Leica Microsystems) in an ice-cold artificial cerebrospinal fluid solution (ACSF), as described previously [37]. Slices were incubated in ACSF at 32–34 °C for 25 min and kept at 22–25 °C thereafter, until transfer to the recording chamber. The osmolarity of all solutions was 300–315 mOsm. Slices were viewed using infrared differential interference contrast optics under an upright microscope (Slice Scope Pro, Scientifica) with a 40 × water-immersion objective.

### Electrophysiology

The recording chamber was continuously perfused (1–2 ml/min) with oxygenated ACSF heated to 32 ± 1 °C using an automatic temperature controller (Warner 278 Instruments). Picrotoxin (100 µM) was added to the solution to block GABA receptor mediated currents. Recording pipettes were pulled from borosilicate glass capillaries (World Precision Instruments) to a resistance of 4–7 MΩ when filled with the intracellular solution. All recordings were conducted with a MultiClamp700B amplifier (Molecular Devices). Intracellular solution contained (in mM): 100 CsCH3O3S, 50 CsCl,3 KCl, 0.2 BAPTA, 10 HEPES, 1 MgCl2, 2.5 phosphocreatine-2Na,2 Mg-ATP, 0.25 GTP-Tris (pH 7.2–7.3 with CsOH, osmolarity 280–290 mOsm). For rectification experiments, dl-AP5 (50 μM) was present in the bath and spermine (100 μM) was added to the intracellular solution. 11 cells from 5 female animals and 9 cells from 3 male animals were used to calculate the rectification index. All sEPSC recordings were conducted in whole-cell voltage-clamp mode (Vh = − 70 mV). Currents were low-pass filtered at 2 kHz and digitized at 20 kHz using a Digidata 1440A acquisition board and pClamp10 software (both from Molecular Devices). Access resistance (10–32 MΩ) was monitored throughout the recordings by injection of 10 mV hyperpolarizing pulses and data were discarded if access resistance changed by > 25% over the course of data acquisition. Cell health and viability was determined through the microscope and recording quality by monitoring the leak current. Recordings with an increase in leak currents more than 20% of the initial target currents were discarded. sEPSCs were detected using an automated sliding-template-based algorithm in pClamp10. This method compares the shape of the detected current to that of a template and has been shown to detect events with amplitude of at least 3 times the square deviation of the noise [38]. All detected events were verified by visual confirmation of a fast rise time and slower exponential decay to baseline. Mean sEPSC amplitude was analyzed from an average sEPSCs trace computed from a minimum of 100 individual sEPSCs. Mean sEPSC frequencies and inter-event intervals were analyzed from 180-s-long trace segments. Evoked responses were triggered by 300-μs constant-current pulses generated by an A310 Accupulser (World Precision Instruments) and delivered at 0.1 Hz via a bipolar tungsten stimulation electrode positioned within 100 μm of the recorded cell. The amplitude of the current pulses was controlled by a stimulus isolator (WPI Linear Stimulus Isolator A395) and was adjusted to elicit monosynaptic responses in the range of 100–300 pA (the required stimulus intensity ranged from 15 to 80 μA). 9 cells from 5 female animals and 14 cells from 7 male animals were used for analysis of sEPSC frequency and amplitude. Recordings were taken from cells within layer V of the infralimbic and prelimbic mPFC.

### Data analysis

All analyses were performed using GraphPad Prism 9 software (GraphPad Software). Data were analyzed using two-tailed Student’s *t*-test, two-way ANOVA with Sidak’s post hoc tests, or Kolmogorov–Smirnov (K–S) as appropriate. Statistical significance for all tests was set at α = 0.05. Experimenters were blind to group conditions when analyzing data for all experiments.

## Results

### Female mice exhibit higher levels of synaptosomal GluA1 and GluA2 expression in the mPFC compared to male mice

**Fig. 1:**
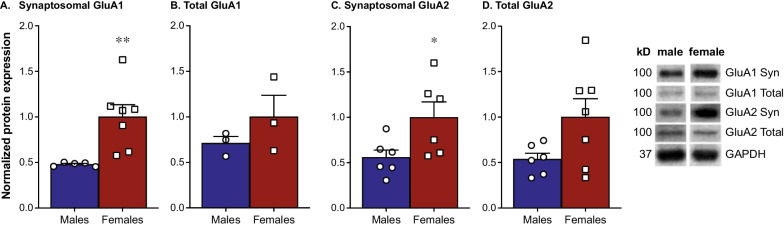
Female mice exhibit higher levels of synaptosomal GluA1 and GluA2 expression in the mPFC compared to male mice. Western blotting revealed higher levels of synaptosomal GluA1 in the mPFC of females compared to males (**A**; *n* = 5–7/group). However, there are no significant differences between females and males in total levels of GluA1 (**B**; *n* = 3/group). We also found females exhibit higher levels of synaptosomal GluA2 in the mPFC compared to males (**C**; *n* = 6/group). Again, these differences are not present in total GluA2, where males and females do not exhibit significant differences (**D**; *n* = 6–7/group)

Baseline levels of synaptosomal and total GluA1 and GluA2 in the mPFC were examined using western blotting. We found females have significantly higher synaptosomal expression of GluA1 than males [Fig. 1A; *t*(10) = 3.237, *p* < 0.01]. This does not extend to total expression of GluA1, as we did not see any significant differences between males and females in this measure [Fig. 1B; *t*(15) = 1.50, *p* = 0.15]. Females also exhibit significantly higher synaptosomal expression of GluA2 than males [Fig. 1C; *t*(10) = 2.351, *p* = 0.04), an effect that does not translate to any significant sex differences in total levels of GluA2 [Fig. 1D; *t*(11) = 2.026; *p* = 0.06].

### Female mice have enhanced glutamatergic transmission in the mPFC compared to male mice

**Fig. 2:**
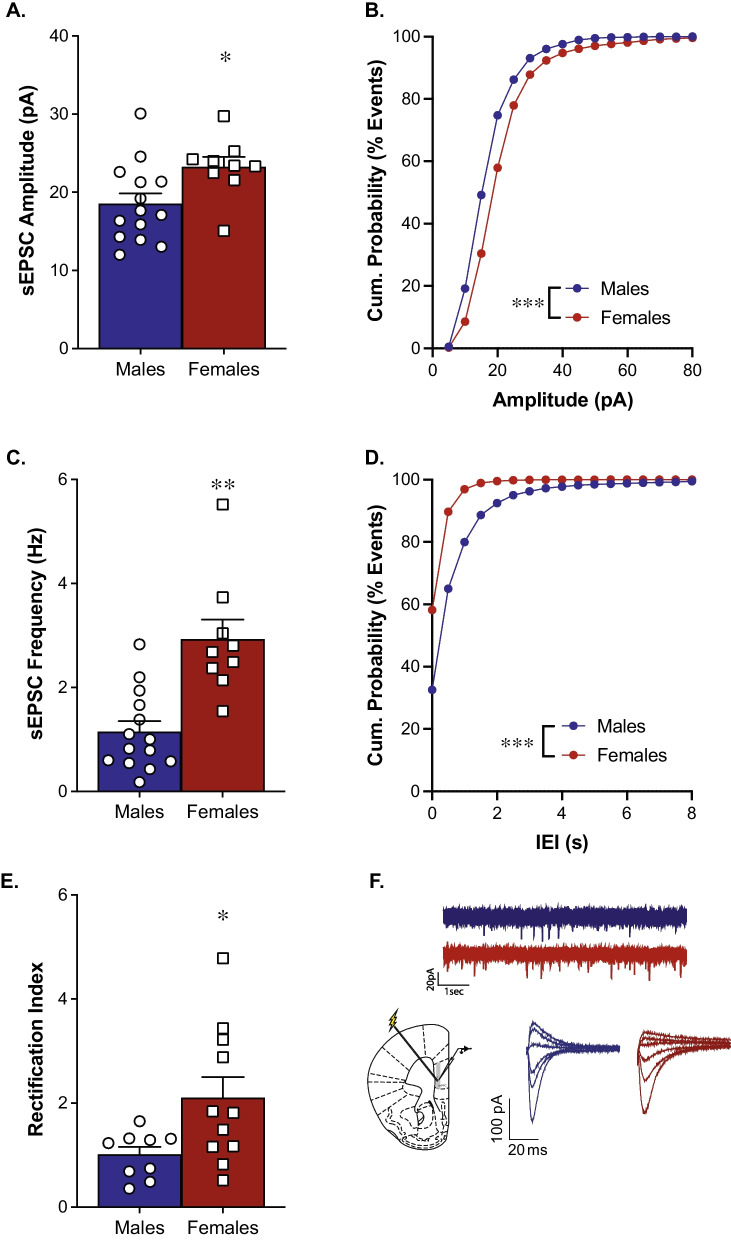
Female mice have enhanced glutamatergic transmission in the mPFC compared to male mice. Whole-cell recordings demonstrate females have heightened sEPSC amplitude (**A**; *n* = 9–14/group) and a rightward shift in the cumulative probability distribution (**B**) compared to males. Females also exhibit heightened sEPSC frequency (**C**; *n* = 9–14/group) and a leftward shift in the cumulative probability distribution of inter-event intervals (**D**) compared to males. Females also exhibit a larger rectification index compared to males (**E**; *n* = 9–11/group). Example electrode placement in the mPFC and representative traces for sEPSC and rectification recordings (**F**)

Baseline glutamate transmission within the mPFC was examined using whole-cell patch clamp recordings. Recordings from female mice revealed significantly higher sEPSC amplitude than males which is further reflected in a rightward shift of the cumulative probability curve [Fig. 2A; *t*(21) = 2.39, *p* = 0.027; Fig. 2B; *p* < 0.001, K–S test]. Females also exhibit significantly higher sEPSC frequency than males, further reflected by a leftward shift in the cumulative probability of inter-event intervals (IEIs) [Fig. 2C; *t*(21) = 4.49, *p* = 0.0002; Fig. 2D; *p* < 0.0001, K–S test]. Females also exhibit a significantly larger rectification index than males, indicating females have more inward rectification in the mPFC than males [Fig. 2E; *t*(18) = 2.375, *p* = 0.03].

## Discussion

Despite established sex differences in the prevalence and presentation of various psychiatric disorders, little is known about the mechanisms driving these differences. The mPFC is an important contributor to psychiatric diseases such as depression, anxiety, and SUD [4], all of which have significant sex differences in clinical presentation. The majority of neurons in the PFC are pyramidal glutamatergic projections [1]. As glutamatergic transmission within the mPFC is implicated in these diseases [4], we investigated whether there could be baseline sex differences in glutamatergic transmission in this region that may underline sex differences in psychiatric disease. Our results demonstrate there is indeed a sex difference in the mPFC, where females exhibit heightened glutamatergic transmission compared to males.

AMPA receptors are the main source of fast excitatory transmission in the central nervous system. There are 4 AMPAR subunits (GluA1-4) that form homo- or heteromers [39, 40]. The various AMPAR subunits are involved in many of the diseases that involve glutamate dysregulation [40–43]. We found females exhibit higher levels of synaptosomal GluA1 and GluA2 in the mPFC compared to males. We do not see any statistically significant sex differences in total GluA1 or GluA2 expression. This would suggest that rather than an overall difference in expression of these subunits, there is greater synaptic AMPA subunit expression. However, there were trends towards higher total protein levels in females suggesting the effects may not be isolated to the synaptosome greater synaptic expression of AMPAR subunits highlights the possibility that females have enhanced glutamatergic transmission within the mPFC compared to males.

Functional differences between GluA1-4 are well-established, with the subunits exhibiting different kinetic properties and distinct roles in synaptic plasticity [44–46]. GluA1 homomers are inwardly rectifying and are proposed to have greater conductance than GluA2-containing heteromers [47–51]. We found that females exhibit a larger rectification index in the mPFC than males. The calculated rectification index in females is also greater than 1, indicating there is more inward rectification in females compared to males. Under our recording conditions, this change in rectification indicates a change in CP-AMPARs. Combined with the kinetic properties and heightened synaptosomal expression of GluA1, we propose this indicates there are more synaptic CP-AMPARs in the mPFC of females compared to males. However, we do see increases in synaptosomal GluA2 along with GluA1, which may suggest overall increases in AMPARs rather than specific increases in CP-AMPARs. As synaptosomal preparations include both membrane bound receptors and intracellular pools, the rectification index measurements more accurately reflect functional differences at the synapse. Overall, these data show there are baseline sex differences in AMPAR distribution within the mPFC. As the calcium-permeable, GluA1 homomers have even higher conductance, this further supports that female mice exhibit greater AMPA transmission than male mice in within this region.

An increased contribution of GluA2-lacking AMPARs is indicative of increased excitatory synaptic strength [52]. Therefore, we investigated whether there are sex differences in excitatory transmission as measured by sEPSC frequency and amplitude. We found that females have a higher sEPSC frequency and larger amplitude in the mPFC compared to males. sEPSC frequency is generally regarded as a measure of presynaptic glutamatergic transmission and amplitude as a measure of postsynaptic glutamatergic transmission. Therefore, the heightened sEPSC frequency and amplitude values we see in females compared to males suggest sex differences in both pre- and postsynaptic glutamate transmission with the mPFC. Overall, our data indicate females have heightened excitatory AMPA transmission in this region that may underlie sex differences in psychiatric disease.

While we uncovered sex differences in excitatory transmission in layer V of the PFC, previously published data demonstrate conflicting findings. In layers V and VI of the prelimbic PFC, males exhibit higher sEPSC amplitudes than females and there were no sex differences seen in sEPSC frequency [30]. The medial PFC is sometimes subdivided into the prelimbic and infralimbic portions and the current study did not differentiate between the prelimbic and infralimbic portions of the mPFC. Therefore, it is possible methodological differences explain this discrepancy. Nonetheless, there are reported aspects of transmission in this region that do not differ between males and females. Maturational trajectories of current–voltage curves, resting membrane potentials, rheobases, mGluR2/3-mediated LTD, and paired pulse ratios in layer V of the PFC are similar between the sexes in rats [53]. Additionally, field excitatory postsynaptic potentials are similar between the sexes across multiple age groups [53]. Together, these data indicate that males and females mature similarly in many aspects of synaptic plasticity within the PFC. As our data indicate female mice have heightened AMPA transmission in this region compared to males, it is likely there are compensatory mechanisms to counteract this difference in transmission.

In line with this hypothesis, the number of action potentials in response to depolarizing steps is lower in adult females than pubescent or juvenile females, an effect of age that is not seen in males [53]. This recapitulates previously published data demonstrating prepubescent females have enhanced excitability in medium spiny neurons within the striatum compared to males [54]. Together, these suggest there may be enhanced excitability in certain regions in the reward system in younger females that decreases over time. As we see heightened AMPA transmission in the PFC of adult females compared to males, it is possible that age-related decreases in cell excitability in the PFC of females serves to balance these changes in AMPA transmission. Additionally, we focused on AMPA expression and function in the current studies, however there may be sex differences in other glutamate receptor subtypes, such as metabotropic glutamate receptors (mGluRs) or N-methyl-d-aspartate receptors (NMDARs). Previous work demonstrated females and males exhibit similar levels of mGluR2/3, mGluR1, and NR2B in the PFC, but females exhibit higher levels of mGluR5 and NR1 than males [55]. Further work could investigate possible sex-specific roles of these receptors in glutamate transmission and cell excitability within the PFC.

Our data indicate there are sex differences in AMPAR expression and function within the mPFC. Aberrant AMPAR expression is thought to underlie a multitude of neuropsychiatric diseases [56]. For example, enhanced AMPAR transmission in the nucleus accumbens is proposed to drive cocaine reinstatement and incubation of cocaine craving [57, 58]. As diseases such as SUD have known sex differences in presentation [18, 24–26, 59], it is possible the sex differences we see in excitatory transmission within the mPFC underlie some of the sex differences seen in diseases such as depression, anxiety, and SUD. Gonadal hormones in both sexes modulate synaptic plasticity in the reward system [25]. As we did not track estrus cycle stage in females in these studies, it is possible the effects we see on excitatory transmission may change with natural fluctuations in gonadal hormone levels. Overall, our data indicate there are baseline sex differences in glutamate transmission that may influence the effectiveness of pharmacotherapies aimed at treating a variety of psychiatric disorders.

### Perspectives and significance

Our data show that glutamatergic transmission within the mPFC is different between male and female mice. We propose that this difference may in part underlie the known sex differences in the prevalence and presentation of psychiatric diseases involving the mPFC. The baseline sex differences we see in excitatory transmission may explain why some treatments for these diseases do not function equally in males and females. Thus, the development of sex-specific pharmacotherapies for the treatment of psychiatric disease may aid in better treatment of psychiatric illness.

## Conclusions

Together, our data demonstrate there are sex differences in excitatory transmission in the mPFC. It is well-established that there are sex differences in the occurrence of diseases such as major depressive disorder and anxiety [19–23]. It is proposed that sex differences in glutamate tone may underlie these differences [33]. Here, we show there are sex differences in glutamate transmission within the mPFC. Further investigation into this is necessary to develop more targeted pharmacotherapies for treatment of psychiatric disease.

## Data Availability

The datasets used and/or analyzed during the current study are available from the corresponding author on request.
